# Efficacy of shoulder arthroscopic surgery for the treatment of rotator cuff injury: A protocol of systematic review and meta-analysis

**DOI:** 10.1097/MD.0000000000020591

**Published:** 2020-06-26

**Authors:** Long-ze Zong, Ming-ming Duan, Wei-wei Yuan, Hao-dong Lu

**Affiliations:** a Department of Joint Surgery; b Department of Trauma Surgery; c Department of Surgical Intensive Care Center, Yanan University Affiliated Hospital, Yan’an, China.

**Keywords:** efficacy, randomized controlled trial, rotator cuff injury, safety, shoulder arthroscopic surgery

## Abstract

**Background:**

This study will investigate the efficacy and safety of shoulder arthroscopic surgery (SAS) for patients with rotator cuff injury (RCI).

**Methods:**

We will systematically search for randomized controlled trials in the electronic databases of PUBMED, EMBASE, Cochrane Library, CINAHL, PsycINFO, Web of Science, Allied and Complementary Medicine Database, Chinese Biomedical Literature Database, and China National Knowledge Infrastructure. All above databases will be searched from their beginning to March 1, 2020 without language restrictions. Two reviewers will independently scan retrieved records, evaluate study quality and extract data. If possible, we will synthesize the data and conduct a meta-analysis by RevMan 5.3 software.

**Results:**

This systematic review will summarize the most recent evidence to explore the efficacy and safety of SAS for patients with RCI.

**Conclusion:**

The findings of this study will help to provide a genuine understanding of perspective from a scientific basis on the efficacy and safety of SAS for patients with RCI.

**PROSPERO registration number:**

PROSPERO CRD42020170009.

## 1 Introduction

Rotator cuff injury (RCI) is associated with severe shoulder pain throughout life,^[1,2]^ which affects millions of people around the world.^[3–5]^ Its prevalence rate is about 20% in general population, and its incidence rate ranges from 10% to 50%.^[6]^ Although the etiology of RCI is still poor understood,^[4,7,8]^ it is essential to develop treatment strategy approach that can benefit patients with RCI.

Numerous studies suggested that shoulder arthroscopic surgery (SAS) can benefit patients with RCI.^[9–24]^ However, evidence from published studies has been conflicting.^[10–24]^ In addition, no previous systematic review exists regarding the efficacy and safety of SAS for the treatment of patients with RCI. This study will investigate the efficacy and safety of SAS in treating RCI.

## 2 Methods and analysis

### 2.1 PROSPERO registration

We registered this study protocol through PROSPERO (CRD42019161502). We have reported it according to the Preferred Reporting Items for Systematic Reviews and Meta-Analysis (PRISRMA) Protocol statement.^[25]^

### 2.2 Study inclusion and exclusion criteria

#### 2.2.1 Types of studies

We will consider all potential randomized controlled trials (RCTs) for inclusion, which explored the efficacy and safety of SAS for patients with RCI. We will not apply restrictions related to the language and publication status.

#### 2.2.2 Types of interventions

In the experimental group, all patients received SAS alone for their interventions.

In the control group, all patients received any treatments without limitations. However, we will exclude any comparators involved in SAS.

#### 2.2.3 Types of participants

We will include any patients who were diagnosed as RCI without restrictions related to the race, age, sex, and severity of RCI.

#### 2.2.4 Types of outcome measurements

The primary outcome is pain intensity of shoulder joint, as measured by Visual Analogue Scale or other scales.

The secondary outcomes are range of shoulder motion (as assessed by Range of Joint Motion Evaluation Chart or other tools), muscle strength of attacked shoulder (as measured by any indexes, such as Cybex Norm isokinetic dynamometer), quality of life (as evaluated by any related scales, such as 36-Item Short Form Survey), and adverse events.

### 2.3 Literature sources and search strategy

We will carry out a systematic search for randomized controlled trials in PUBMED, EMBASE, Cochrane Library, CINAHL, PsycINFO, Web of Science, Allied and Complementary Medicine Database, Chinese Biomedical Literature Database, and China National Knowledge Infrastructure. We have retrieved all electronic databases from inception to March 1, 2020 without language restrictions. The detailed search strategy utilized in PUBMED is created (Table 1). Similar search strategies will be adapted to other electronic databases.

**Table 1:**
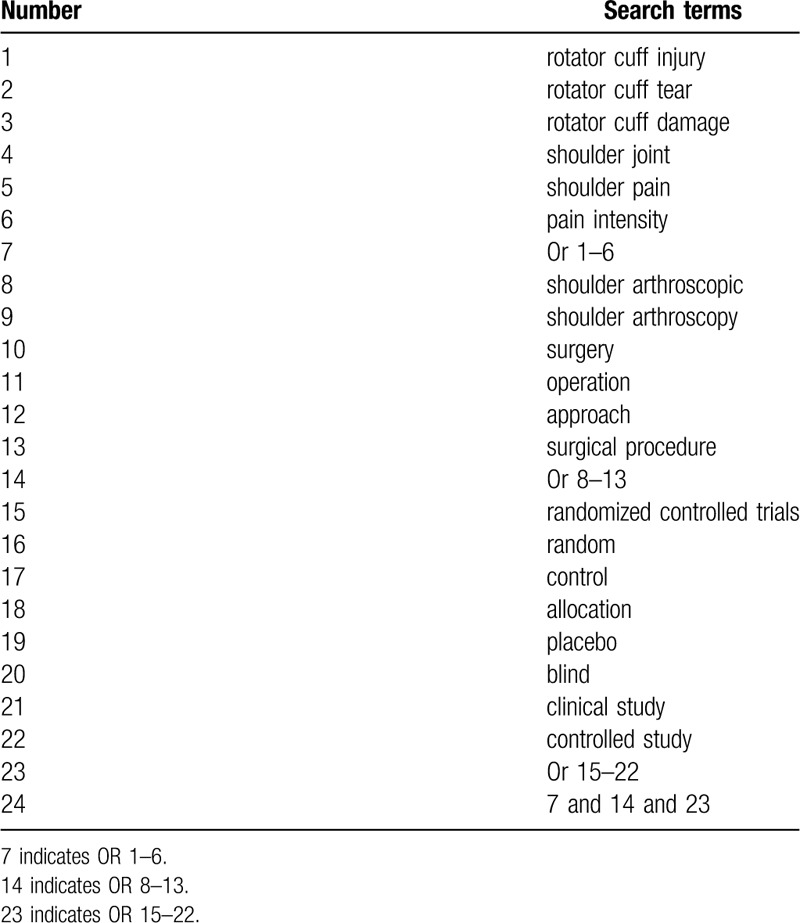
Search strategy of PUBMED.

Besides, we will also search dissertations, conference proceedings, and reference lists of relevant reviews.

### 2.4 Data collection and analysis

#### 2.4.1 Selection of studies

Titles and abstracts of all retrieved records will be screened, and unrelated studies will be removed. Then, full text of all potential articles will be read carefully to determine whether they are eligible against all inclusion criteria. The excluded studies will be listed in a table with clear reasons. The process of study selection will be presented in a PRISMA flowchart. Two independent reviewers will conduct all study selection, respectively. Any different views between both of them will be solved by a third reviewer through discussion.

#### 2.4.2 Data extraction and management

All data will be collected from included trials by 2 independent reviewers using a standardized data collection sheet. Any confusion between both of them will be solved by discussion with a third reviewer involved. The extracted information is publication information (such as title, first author, and publication year), participant characteristics (such as age, sex, and severity of RCI), study methods (such as details of randomization, allocation, and blind), specifics of interventions and controls (such as intervention types, dosage, and frequency), outcomes, adverse events, and other relevant information.

#### 2.4.3 Missing data dealing with

Any missing or insufficient information will be requested from primary trial authors. If we cannot obtain it, we will analyze available data only, and will discuss its impacts to the study findings.

### 2.5 Study quality assessment

Two reviewers will independently appraise study quality for all included RCTs by Cochrane risk of bias tool through 7 different aspects. Each area is rated as high, unclear, and low risk of bias. If we will encounter any differences between two of them, a third reviewer will help to reach a consensus by discussion.

### 2.6 Statistical analysis

We will carry out statistical analysis by RevMan 5.3 software. All continuous values will be calculated as weighted mean difference or standard mean difference and 95% confidence intervals (CIs), and all dichotomous values will be exerted as risk ratio and 95% CIs. *I*^2^ statistic will be tested to identify potential heterogeneity among eligible RCTs. We define it as follows: *I*^2^ ≤50% suggests homogeneity, and a fixed-effects model will be applied; *I*^2^ > 50% indicates considerable heterogeneity, and a random-effects model will be utilized. If it is possible, we will conduct a meta-analysis. As for obvious heterogeneity, we will perform a subgroup analysis to detect its sources. If necessary, we will carry out a narrative summary.

### 2.7 Additional analysis

#### 2.7.1 Subgroup analysis

We will perform a subgroup analysis to explore sources of obvious heterogeneity according to the variations in study and patient characteristics, study methods, interventions and controls, and outcomes.

#### 2.7.2 Sensitivity analysis

We will carry out a sensitivity analysis to examine the robustness of study results by eliminating low quality studies.

#### 2.7.3 Reporting bias

We will perform funnel plot and Egger regression test to identify reporting bias if at least 10 eligible RCTs are included.^[26,27]^

### 2.8 Ethics and dissemination

This study will not require ethical documents because it will not obtain individual patient data. This study expects to be published at a peer-reviewed journal.

## 3 Discussion

Studies suggest that SAS may benefit patients with RCI; however, the evidence from RCTs is inconsistent. In addition, we do not identify any published systematic review addressing this issue. With an increasing number of clinical trials, this proposed study aims to assess the efficacy and safety of SAS for the treatment of RCI. It will summarize the up-to-date evidence of SAS for the treatment of RCI. This study will provide evidence to determine whether SAS is effective and safe for patients with RCI, which may benefit both clinical practice and further studies.

## Author contributions

**Conceptualization:** Ming-ming Duan, Hao-dong Lu.

**Data curation:** Long-ze Zong, Wei-wei Yuan, Hao-dong Lu.

**Formal analysis:** Long-ze Zong, Ming-ming Duan, Hao-dong Lu.

**Investigation:** Hao-dong Lu.

**Methodology:** Long-ze Zong, Ming-ming Duan.

**Project administration:** Hao-dong Lu.

**Resources:** Long-ze Zong, Ming-ming Duan, Wei-wei Yuan.

**Software:** Long-ze Zong, Ming-ming Duan, Wei-wei Yuan.

**Supervision:** Hao-dong Lu.

**Validation:** Long-ze Zong, Ming-ming Duan, Wei-wei Yuan, Hao-dong Lu.

**Visualization:** Long-ze Zong, Ming-ming Duan, Wei-wei Yuan, Hao-dong Lu.

**Writing – original draft:** Long-ze Zong, Wei-wei Yuan, Hao-dong Lu.

**Writing – review & editing:** Long-ze Zong, Ming-ming Duan, Wei-wei Yuan, Hao-dong Lu.
